# Child Crime as a Result of War

**Published:** 1917-05

**Authors:** 


### Child Crime as a Result of War.

According to the *Literary Digest*, a sad increase of crime among German children betwen the ages of twelve and eighteen is disclosed by Dr. Albert Hellwig in his recent book on "The War and Crime Among the Young," published in German and reviewed in the London *Daily Mail.* The German author is police-court judge who, to get material for his work, "scoured German newspapers, obtained numerous reports from institutions and societies which care for the young, and finally he wrote direct to the police authorities in several hundred German towns." The last proof-sheets of the book went to press on June 27, 1916, we are told, so that almost exactly two years of war are covered. In our issue for July 8, 1916, we cited Judge Hellwig's article on the subject in *Grenzboten* (Berlin), where some generalizations were offered on the theme that now is presented with startling particulars. The British reviewer notes these details in his summary of the German work:

"In Berlin in 1915 there were twice as many crimes among the young as in 1914. Munich reported that in the first three months of 1915 the number of young criminals equaled the total for 1914. From Stuttgart came the news that crime had very considerably increased, especially among those of school age. Frankfort rejoiced in a decrease of 55 per cent of minor offenses but an increase of 40 per cent in serious crimes.

"The German author is amazed at a long list of such reports, especially as hundreds of thousands of youths aged from sixteen to eighteen were not at home but in the German army as volunteers.

"His conclusion is: 'From all these figures it is evident that crime among the young diminished in some places during the first months of the war. But afterward the increase was all the greater, at least in the larger towns, and as regards crimes tried before a judge and jury.' "

A few specimen crimes are selected:

"A servant girl aged fifteen was tried by court martial at Greifswald and was sentenced to three years for setting fire to a granary. Two Potsdam youths who waylaid a beer wagon and battered the driver senseless with his own beer bottles obtained £11 16s. in loot. In Munich on May 1, 1915, a nine-year-old boy killed his sister, aged one year, by cutting her throat. At Oels (Silesia) a boy of seventeen heard in December, 1915, that a neighbor (her husband serving in the army) had sold her horse and had the money at home. He went there, but the woman was out. After stealing the money he fetched an ax and murdered three children who had witnessed the theft. His arrest followed, but during the whole trial he showed not the slightest regret for the crime. His sentence was the highest possible—fifteen years' penal servitude.

"In Berlin, during the same month, Helene P——— killed her sister with a kitchen knife in order to steal her savings. At Hamburg, on July 14, 1915, two girls, aged, respectively, seventeen and fifteen, entered the dwelling of a woman in the Elsastrasse, murdered the woman, and stole what they could find. And so on through scores of pages *ad nauseam!*"

Crimes of sheer brutality seem to show the greatest increase, for which Dr. Hellwig is said to give the following reasons:

"(a) Economic conditions—poverty in the first period of the war and high wages afterward; (b) anti-educational influences —fathers absent from home, slackening of school discipline, trashy war books and films; (c) fewer policemen, caused by the mobilization, and the amnesty for youthful criminals decreed in the early days of the war."

Though the author is very discreet on this point, for he is a paid servant of the State, the British reviewer finds a conviction that the "hate campaign" is an important cause of the increase of youthful criminality. As we read in *The Daily Mail:*

"It appears that certain German circles agitated against the inculcation of hate, suggesting that the schools should be employed to spread better influences. This called forth a decree (*Erlass*) from the Prussian government on January 15, 1916, in these terms: 'Wishes have been expressed recently that the teachers in our schools should combat by suitable instruction the spread and deepening of national hate and pave the way for the future reconciliation of civilized nations. No opportunities may be permitted for such endeavors, which are inspired by the feelings of universal brotherhood and international peace-piffle."

"Dr. Hellwig could not easily fly in the case of this decree, but he works round it. The last six pages of his chapter on 'Causes' are devoted to hate. He writes:

"'The excessive excitement of the childish imagination by the events of the war, especially as they are depicted in trashy literature, is one of the brutalizing influences acting on our young people in war time. To inoculate the children with hate *would* breed lust for revenge, and *could* only bear evil fruit.'

"The italics are mine, and are meant to show how Dr. Hellwig's position compels him to write in a hypothetical sense, but he leaves only one inference open—viz., that hate has been one of the main causes of the increased criminality among German children during the war."

